# Effects of thoracic epidural anesthesia on survival and microcirculation in severe acute pancreatitis: a randomized experimental trial

**DOI:** 10.1186/cc13142

**Published:** 2013-12-05

**Authors:** Kai A Bachmann, Constantin JC Trepte, Lena Tomkötter, Andrea Hinsch, Jan Stork, Wilken Bergmann, Lena Heidelmann, Tim Strate, Alwin E Goetz, Daniel A Reuter, Jakob R Izbicki, Oliver Mann

**Affiliations:** 1 Department of General-, Visceral- and Thoracic Surgery, University Medical Center Hamburg-Eppendorf, Martinistr. 52, D-20246 Hamburg, Germany; 2 Center for Anesthesiology and Intensive Care Medicine, University Medical Center Hamburg-Eppendorf, Martinistrasse 52, D-20246 Hamburg, Germany; 3 Department of Pathology, University Medical Center Hamburg-Eppendorf, Martinistr. 52, D-20246 Hamburg, Germany

## Abstract

**Introduction:**

Severe acute pancreatitis is still a potentially life threatening disease with high mortality. The aim of this study was to evaluate the therapeutic effect of thoracic epidural anaesthesia (TEA) on survival, microcirculation, tissue oxygenation and histopathologic damage in an experimental animal model of severe acute pancreatitis in a prospective animal study.

**Methods:**

In this study, 34 pigs were randomly assigned into 2 treatment groups. After severe acute pancreatitis was induced by intraductal injection of glycodesoxycholic acid in Group 1 (n = 17) bupivacaine (0.5%; bolus injection 2 ml, continuous infusion 4 ml/h) was applied via TEA. In Group 2 (n = 17) no TEA was applied. During a period of 6 hours after induction, tissue oxygen tension (tpO_2_) in the pancreas and pancreatic microcirculation was assessed. Thereafter animals were observed for 7 days followed by sacrification and histopathologic examination.

**Results:**

Survival rate after 7 days was 82% in Group 1 (TEA) versus 29% in Group 2: (Control) (*P* <0.05). Group 1 (TEA) also showed a significantly superior microcirculation (1,608 ± 374 AU versus 1,121 ± 510 AU; *P* <0.05) and tissue oxygenation (215 ± 64 mmHg versus 138 ± 90 mmHG; *P* <0.05) as compared to Group 2 (Control). Consecutively, tissue damage in Group 1 was reduced in the histopathologic scoring (5.5 (3 to 8) versus 8 (5.5 to 10); *P* <0.05).

**Conclusions:**

TEA led to improved survival, enhanced microcirculatory perfusion and tissue oxygenation and resulted in less histopathologic tissue-damage in an experimental animal model of severe acute pancreatitis.

## Introduction

Severe acute pancreatitis (SAP) is a life threatening disease with a high mortality despite improved treatment strategies. The incidence of SAP has increased during the last decades [1,2]. Progression from the mild edematous to the hemorrhagic necrotizing form determines outcome [3-8]. Up to now, no causal treatment of pancreatitis is known. Although the pathophysiologic cascade of its development and progress is poorly understood, microcirculatory disturbances are considered to be a key factor [9,10].

The rationale of this trial is based on the generally accepted finding that an improvement of pancreatic microcirculation prevents the progression from mild edematous to severe necrotizing pancreatitis [3-7,11]. Different therapeutic interventions for improving pancreatic microcirculation have been evaluated in the last years [9,12-16]. Therapeutic efforts aim at saving injured tissue from infarction and necrosis by improving microcirculatory perfusion and oxygen supply. The main idea in our approach is sympathetic block by thoracic epidural anesthesia (TEA) with consecutive redistribution of blood flood toward the splanchnic vessels [17]. This effect could be demonstrated in various trials [18-21]. In perioperative and experimentally induced hemorrhage positive effects due to epidural anesthesia on gastrointestinal microcirculation could be demonstrated [22,23]. TEA is further reported to improve renal and gastrointestinal perfusion during endotoxemia [22,24,25]. This is also true for microvascular blood flow in the liver or ileal mucosa in other models of systemic inflammation [24,26]. Some first attempts also showed promising results for the use of TEA in pancreatitis demonstrating reduced liver injury, improved ileal mucosal capillary perfusion and survival as well as pancreatic microcirculation in the rat [26-28].

Another aspect is,that microcirculation is also dependent on macrocirculatory conditions and an adequate macrocirculation is an indispensible precondition. For evaluation of macrocirculatory conditions the use of advanced hemodynamic monitoring has substantially increased in recent years in intensive care and perioperative settings [29]. In part, results of this study have already been reported demonstrating the impact of two different treatment strategies for early goal-directed fluid therapy [30]. However, to date no data and final validation exist evaluating the therapeutic effect of TEA in SAP during controlled hemodynamic conditions. We hypothesized that improvement of microcirculation due to redistribution of blood flow in the inflamed tissue by TEA would result in improved pancreatic microcirculatory conditions and outcome in SAP.

Therefore, the aim of this study was to evaluate the therapeutic effects of TEA in an experimental model of SAP. The primary outcome variable was survival after seven days. Secondary outcome variables were pancreatic microcirculation and tissue oxygenation during the first six hours after induction as well as the extent of histopathologic tissue-damage.

## Materials and methods

The study was approved by the Governmental Commission on the Care and Use of Animals of the City of Hamburg. The animals received care in compliance with the ‘Guide for the Care and Use of Laboratory Animals’ (NIH publication No. 86–23, revised 1996) and experiments were carried out according to the ARRIVE guidelines [31].

### Study design

The study was designed as a randomized trial in 34 German domestic pigs (German Hybrid). Animals were randomized to two different treatment groups: Group 1 (TEA, n = 17) received TEA after induction of SAP. In Group 2, acute pancreatitis was induced, however no TEA was performed (Control; n = 17).

### Anesthesia and surgical preparation

After fasting overnight, ketamine (10 mg/kg), azaperone (4 mg/kg), midazolam (15 mg) and atropine (0.0015 mg/kg) were administered for premedication. After preoxygenation, induction of anesthesia and orotracheal intubation were performed. Continuous infusion of fentanyl (0.05 mg/kg/hour) and sevoflurane (Fet 2.0) were used for balanced anesthesia. The animals were mechanically ventilated using tidal volumes of 10 ml/kg. Inspiratory oxygen fraction (FiO_2_) was set at 0.35 and respiratory rate was adjusted to maintain end expiratory pCO2 at 35 to 40 mmHg (Zeus, Draeger Medical Systems, Lübeck, Germany). Moreover, a gastric tube was brought into position. For monitoring of heart rate and oxygen saturation a 5-lead electrocardiogram and pulse oximetry were used. Body temperature was kept constant using forced-air warming blankets.

**Figure 1:**
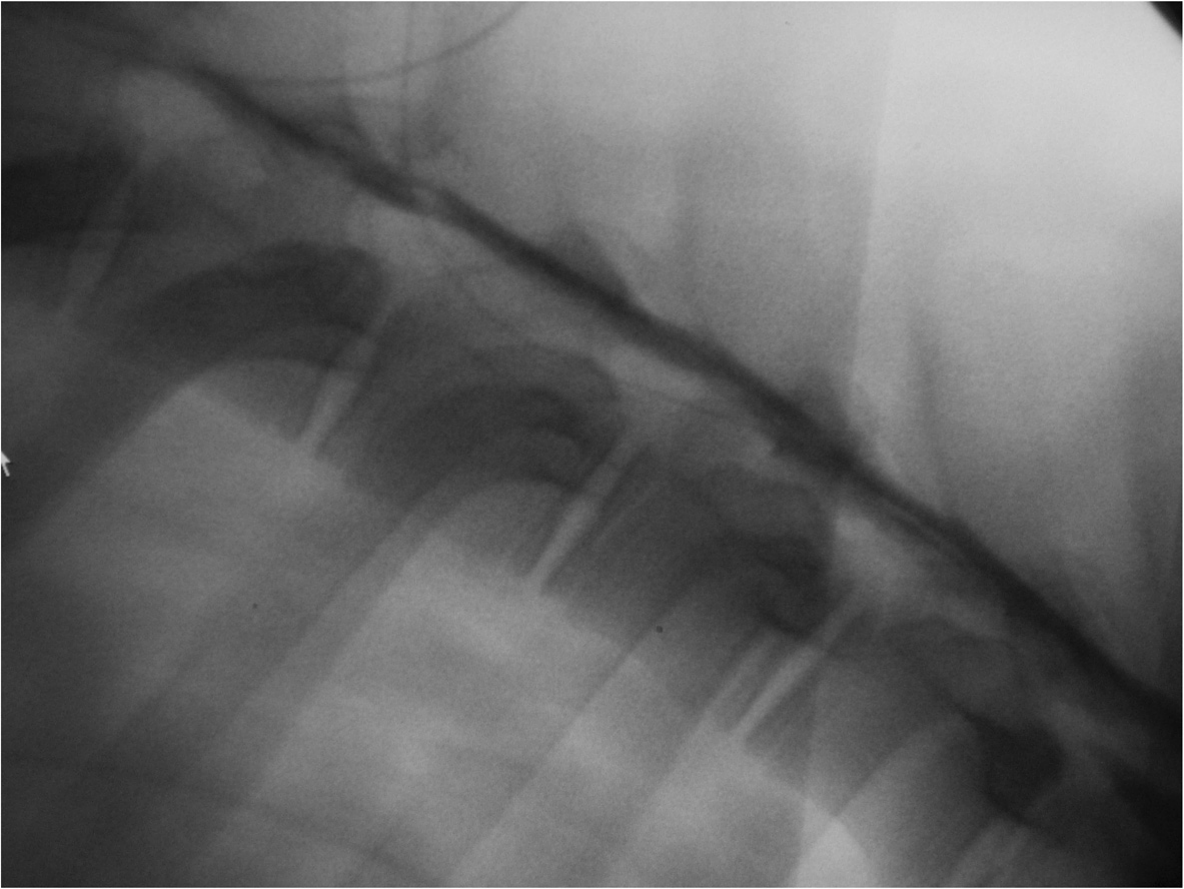
Epidurogram after placement of the epidural catheter.

Prior to the beginning of further surgical preparation, animals randomized to Group 1 (TEA) were positioned on the right side and a thoracic epidural catheter was introduced between Th 7 and Th 8 under sterile conditions and radiographic control. The catheter was advanced 2 cm into the epidural space and correct positioning of the catheter was verified and documented by an epidurogram as gold standard (Figure 1). For these purposes 5 ml of contrast agent were injected in the epidural space during fluoroscopy to exclude an intravascular position of the catheter. Thereby, it was verified that a minimal spread over 6 to 8 segments was present and no misplacement of the epidural catheter had occurred.

All animals were placed in the supine position and after thorough disinfection and sterile coverage, the femoral artery was canulated using a 5 F thermistor tipped arterial catheter (PICCO, PV 2015 L20, Pulsion, Feldkirchen, Germany). Two central venous catheters were surgically introduced into the internal and external jugular vein, one for volume administration, the other to enable injection of cold indicator for transcardiopulmonary thermodilution. Hemodynamic data were recorded using a PiCCOplus monitoring system (version 6.0, Pulsion Medical Systems, Munich, Germany).

Thereafter, a transverse upper laparotomy was performed. A urinary catheter was placed directly into the bladder for urinary drainage and intraoperative accounting of urine. Pancreas and duodenum were mobilized and fixed at the laparotomy incision for intraoperative measurements, whereupon meticulous attention was paid to strainless positioning. After dissection and cannulation of the main pancreatic duct (Vasofix 0,8 mm, B. Braun, Melsungen, Germany) between the pancreas and duodenal wall, a flexible polarographic measuring probe (CCP1, Licox, Kiel, Germany) was placed in the pancreatic head for continuous measurement of the tissue oxygen tension (tpO2) [32,33]. A Laser-Doppler imager (Laser-Doppler Imager LDI2, Moor, Millway, UK), was installed to assess microcirculation in the pancreatic head. The laser is scattered by the tissue and moving blood cells in the capillaries, arterioles and venules. The moving blood cells cause frequency shifts that are processed to produce a color coded map of the scanned area. The Laser-Doppler imager was positioned above the pancreas and the region of interest (pancreas) was marked in a color coded map of the corresponding image. The mean microcirculation in the region of interest was calculated automatically [9].

### Hemodynamic management

**Figure 2:**
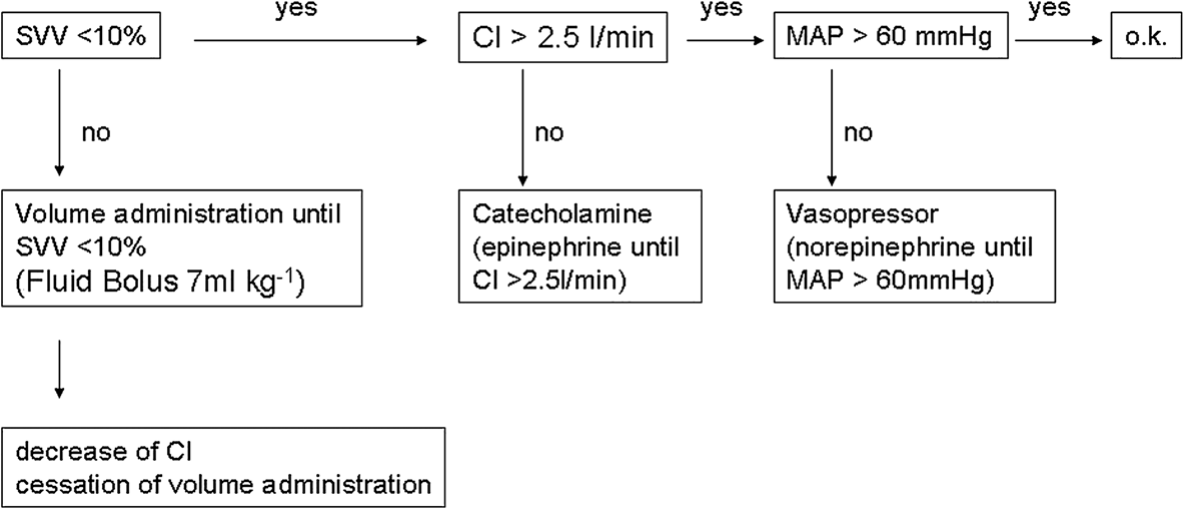
**Algorithm for guidance of fluid therapy.** Stroke volume variation (SVV), Cardiac Index (CI), mean arterial pressure (MAP).

In both groups hemodynamic management was carried out according to a defined treatment algorithm [30]. Ringer’s solution and hydroxyethylstarch 6% 130/0.4 were administered at a fixed ratio of 2:1. The treatment algorithm for guidance of fluid therapy is presented in Figure 2. Hemodynamic data were assessed continuously during the entire procedure as well as with each point of measurement.

### Therapeutic intervention

Animals in Group 1 (TEA, n = 17) received TEA after induction of acute pancreatitis. Initially, a bolus of 2 ml bupivacaine 0.5% was given via the epidural catheter, followed by a continuous application at a fixed rate of 4 ml/hour using an automated infusion pump (Pega® Plus, Venner Medical, Kiel Germany). We aimed for blocking three to four segments above and below the site of insertion.

In group 2 (control; n = 17), acute pancreatitis was induced while no TEA was performed.

### Measurement and experimental protocol

After an initial equilibration, baseline measurements (M0) were performed. Measurements included blood gas analysis, measurement of tpO2 and microcirculation in the pancreatic head as well as thermodilution measurements for assessment of hemodynamic conditions. After completion of baseline measurements, in both groups SAP was induced by intraductal infusion of 0.8 ml/kg glycodesoxycholic acid (10 mmol/L, pH 8, Sigma, Steinheim, Germany) during a period of 15 minutes as previously described, using an automated infusion system (Perfusor® fm (MFC), B Braun, Melsungen, Germany) to ensure a standardized infusion pressure and avoid pancreatic pressure necrosis [4,9,30,34]. Thereafter, the cannula was removed and the pancreatic duct was ligated. Fifteen minutes (M1) and 75 (M2) minutes after completion of the intraductal infusion, measurements were repeated. After M2 in animals randomized to Group 1 (TEA) a bolus of bupivacaine was applied via the epidural catheter, immediately followed by a continuous application at a fixed rate as described. After the beginning of the therapeutic intervention, measurements were repeated every 60 minutes (M3 to M8). After completion of the intraoperative measurements (M8), all catheters were removed except for the TEA catheter in Group 1 (TEA) as well as a central line, tunneled to the dorsal neck, for application of analgesic medication and blood gas sampling in the postoperative course. The abdominal cavity and incision of the neck were closed and anesthesia was ceased. The animals were extubated and transferred to heated boxes in the animal facility. In animals randomized to Group 1 (TEA) the infusion pump for application of bupivacain via the epidural catheter during the postoperative observation interval was attached to the back using a special dressing.

### Survival and postoperative observation

Animals were closely monitored for seven days. Analgesics were given every six hours (piritamide 15 mg). Once a day blood samples for evaluation of pancreatic amylase, total bilirubin (TBIL), liver enzymes (aspartate-transaminase (AST), alanine-transaminase (ALT)), leucocyte count, lactate, creatinine, prothrombin time (PT) and partial thromboplastin time (pTT) were taken via the central venous catheter and laboratory analysis was carried out. Hemoglobin, leucocytes and thrombocytes were counted with a fully automatic blood count analysis unit. TBIL was measured by spectral absorption while the measurement of ALT and AST was based on their enzyme activity. The concentration of amylase was measured by an amylase activity enzyme kit (Abcam, Cambridge, UK). Isotope dilution mass spectrometry was applied to analyze serum creatinine. PT and pTT were measured in a fully automated system as well. Moreover, porcine well-being was assessed in all animals [35].

Animals surviving the observation period were re-anesthetized on the seventh postoperative day, and sacrificed by fast injection of potassium chloride during deep anesthesia. The correct positioning of the thoracic epidural catheter was reconfirmed by another epidurogram. Postmortem examination was carried out and the pancreas was removed for histopathologic examination. In animals that died during the postoperative observation period postmortem examination, removal of the pancreas and the epidurogram were conducted immediately after diagnosis of death. Specimens of the pancreas were stored in 3.5% buffered formalin and routinely processed. They were embedded in paraffin and 5 μm slices were stained with hematoxylin and eosin. The slices were examined by an experienced pathologist blinded to the treatment (AH) using an established scoring system [9,30]. The median score of the histopathologic examination is based on the analysis of 10 high power fields (HPF) in four different slices.

### Power calculation and statistical analysis

The primary endpoint of the trial was survival. Secondary endpoints were pancreatic microcirculation, tissue oxygenation and histopathologic tissue damage. A detectable difference of 25% versus 75% in survival was used to calculate group size. The calculated group size (using 5% alpha error and 80% power) was n = 17. SPSS® for Windows® (Version 13.0) (SPSS Inc., Chicago, IL, USA) was used for statistical analysis. Survival curves were plotted using the Kaplan-Meier method and data were analyzed using the log-rank test. Assessment of normal distribution was conducted using the Kolmogorov-Smirnov-Test. Descriptive analysis of parametric parameters is expressed as means and standard deviation. Ordinal data were expressed as median and range. For analysis of the difference between the groups in repeated measurements the variance analysis for repeated measurements (ANOVA) followed by a time-by-treatment-interaction test was used. Additionally, the area under the curve was calculated during the intraoperative treatment interval (M2 to M8). Differences between the treatment groups were analyzed using a one way ANOVA. Significance statements refer to *P* values of two-tailed tests that were less than 0.05.

## Results

In both groups, 17 animals were studied. There were no statistically significant differences between the different treatment groups. Mean body weight of the animals in Group 1 (TEA) was 32.0 ± 7.5 versus 32.0 ± 6.9 kg in Group 2 (Control), while mean body length was 102 ± 5 cm and 102 ± 6 cm (*P* >0.05).

### Survival and postoperative observation

**Figure 3:**
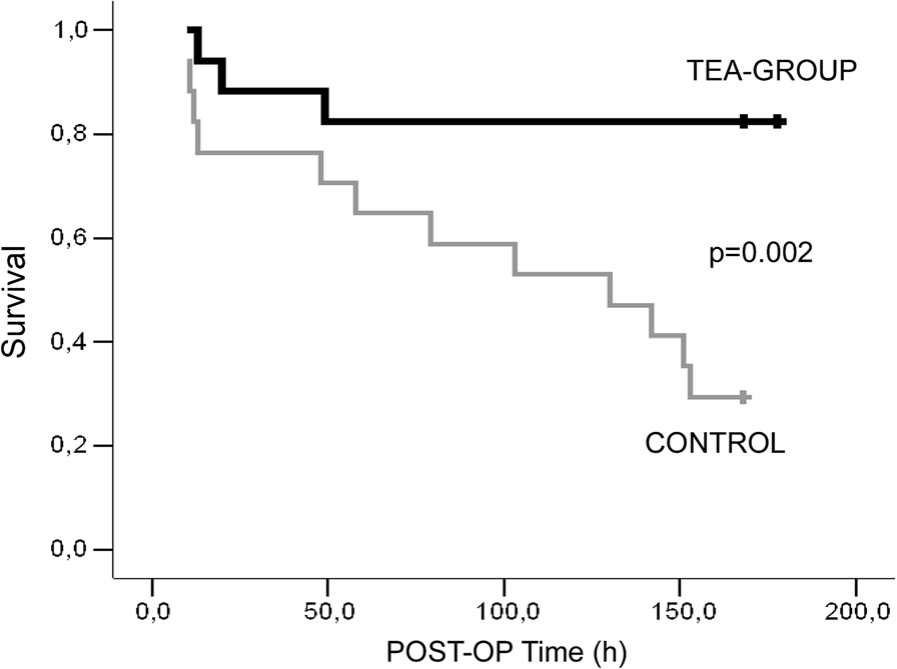
Kaplan Meier curve for survival of animals in the postoperative treatment interval (TEA Group versus Control-Group).

Overall survival after seven days was 82.4% in Group 1 (TEA) versus 29.4% in Group 2 (Control) (*P* <0.001). Survival data are presented in Figure 3.

During the postoperative course, evaluation of the ‘Porcine Well-being Score’ also demonstrated significantly better results in Group 1 (TEA) (Table 1).

**Table 1:** Scoring results of the porcine wellbeing score

**Porcine wellbeing score**	**Day 1**	**Day 2**	**Day 3**	**Day 4**	**Day 5**	**Day 6**	**Day 7**
**TEA**	34 ± 13	35 ± 17	34 ± 17	35 ± 17	37 ± 18	37 ± 18	36 ± 18
**Control**	28 ± 17	29 ± 20	27 ± 21	25 ± 22	23 ± 23	17 ± 22*	14 ± 22*

*Represents statistically significant difference (*P* <0.05). Data are presented as mean ± standard deviation. (Scoring Range: 0 to 50).

### Tissue oxygenation and microcirculation of the pancreas

**Figure 4:**
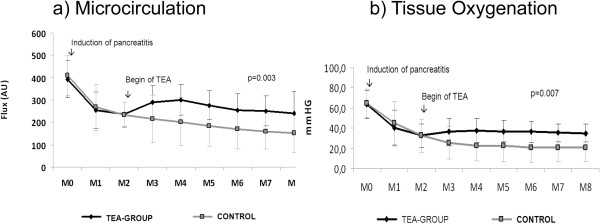
**Microcirculation and tissue oxygenation (tpO2). a)** Pancreatic microcirculation and **b)** tissue oxygenation in the pancreatic head. (MP0 = before induction of the acute pancreatitis, MP1 and 2 after induction of the acute pancreatitis, MP3 to 8 during TEA. Mean and standard deviation; Value in Flux (Arbitrary Unit (AU)).

In both groups (groups 1 and 2), strong decreases of pancreatic microcirculation (Flux) and tpO2 were detected after induction of SAP. After the beginning of TEA, pancreatic microcirculation improved in Group 1 (TEA) and a significant time-by-treatment interaction was detected in the variance analysis for repeated measurements (*P* <0.001). Comparing the areas under the curve during the treatment interval a significantly enhanced pancreatic microcirculation was found in Group 1 (TEA) (1,608.4 ± 374.4) versus Group 2 (Control) (1,121.0 ± 510.) (*P* = 0.003) (Figure 4). Concerning the tissue oxygenation, a significant time-by-treatment interaction was detected in the variance analysis for repeated measurements (*P* <0.001) and when comparing the areas under the curve during the treatment interval a significantly better tissue oxygenation was found in Group 1 (Group 1 (TEA) 215 ± 64 in comparison to Group 2 (Control) 138 ± 90; *P* = 0.007) (Figure 4). The detailed course is presented in Table 2.

**Table 2:** Data on microcirculation, tissue oxygenation, hemodynamics, blood gas analysis and fluid balance

**Parameter**	**Group**	**M 0**	**M 1**	**M 2**	**M 3**	**M 4**	**M 5**	**M 6**	**M 7**	**M 8**
**Tissue oxygenation(tpO**_**2**_**mmHg)**	**1 (TEA)**	63.8 ± 14.1	40.2 ± 17.7	32.5 ± 11.4	36.3 ± 13.7	37.1 ± 12.2	36.8 ± 11.0	36.2 ± 10.5	35.2 ± 9.1	35.0 ± 9.1
	**2 (Control)**	64.2 ± 14.1	45.2 ± 21.4	32.4 ± 16.2	25.7 ± 15.9*	22.8 ± 15.6*	22.2 ± 15.9*	20.4 ± 15.0*	20.5 ± 14.3*	20.4 ± 14.2*
**Microcirculation(Flux AU)**	**1 (TEA)**	391.9 ± 82.8	253.3 ± 81.2	236.1 ± 55.1	288.9 ± 73.4	300.4 ± 68.5	276.2 ± 65.7	254.1 ± 73.8	249.9 ± 66.0	241.5 ± 97.0
	**2 (Control)**	408.5 ± 88.3	267.4 ± 103.4	233.2 ± 56.3	215.9 ± 104.9*	201.8 ± 108.1*	182.4.0 ± 90.0*	168.6 ± 89.0*	160.5 ± 80.7*	150.6 ± 85.7*
**HR (min**^**-1**^**)**	**1 (TEA)**	90.5 ± 24.9	91.9 ± 24.5	95.4 ± 21.5	94.4 ± 19.9	97.7 ± 16.8	100.7 ± 16.3	99.4 ± 14.6	99.3 ± 10.3	99.4 ± 13.2
	**2 (Control)**	85.5 ± 13.8	85.4 ± 14.9	91.1 ± 16.6	95.5 ± 16.7	98.9 ± 15.0	101.0 ± 13.8	103.3 ± 14.7	102.6 ± 16.5	100.8 ± 11.3
**MAP (mmHg)**	**1 (TEA)**	72.5 ± 10.6	74.1 ± 11.5	76.8 ± 10.6	74.8 ± 8.7	74.9 ± 8.4	70.9 ± 7.2	67.2 ± 6.6	64.1 ± 5.3	62.2 ± 6.2
	**2 (Control)**	74.7 ± 10.4	73.3 ± 8.3	73.5 ± 9.4	73.4 ± 7.7	73.1 ± 11.2	69.6 ± 5.8	66.5 ± 6.7	64.2 ± 6.2	61.9 ± 6.8
**CI (L/min)**	**1 (TEA)**	4.7 ± 1.3	4.9 ± 1.3	5.2 ± 1.1	5.1 ± 0.9	5.0 ± 0.9	5.2 ± 0.9	4.8 ± 0.7	4.6 ± 0.5	4.7 ± 0.8
	**2 (Control)**	4.6 ± 0.8	4.6 ± 0.8	5.1 ± 1.1	5.1 ± 0.8	5.1 ± 0.9	5.1 ± 0.9	5.2 ± 1.0	5.2 ± 1.1	*5.2* ± 0.9
**SVV (%)**	**1 (TEA)**	6.5 ± 2.7	6.2 ± 1.1	5.9 ± 1.0	5.9 ± 1.0	7.1 ± 1.9	7.1 ± 1.5	6.5 ± 1.5	7.1 ± 1.5	7.3 ± 1.5
	**2 (Control)**	6.6 ± 1.6	6.8 ± 1.6	6.1 ± 1.3	6.1 ± 1.3*	6.9 ± 1.8*	6.6 ± 0.9*	6.2 ± 1.6*	6.8 ± 1.7*	7.2 ± 2.6*
**CVP (mmHg)**	**1 (TEA)**	6.7 ± 1.9	6.6 ± 1.7	7.0 ± 1.9	6.6 ± 2.3	6.5 ± 2.2	6.4 ± 1.7	6.6 ± 1.8	6.7 ± 1.6	6.6 ± 1.9
	**2 (Control)**	7.2 ± 2.4	6.6 ± 1.9	7.1 ± 2.0	7.0 ± 1.7	7.1 ± 2.0	7.2 ± 2.2	7.8 ± 2.3	7.5 ± 2.6*	8.1 ± 2.9*
**p**_**a**_**O**_**2**_**(mmHg)**	**1 (TEA)**	161.1 ± 67.7	147.8 ± 24.9	139.1 ± 14.7	137.1 ± 12.7	134.7 ± 16.5	131.1 ± 17.6	130.2 ± 18.0	126.8 ± 17.2	125.3 ± 17.3
	**2 (Control)**	159.2 ± 21.2	145.4 ± 16.8	141.1 ± 14.9	138.6 ± 16.7	128.8 ± 12.7	124.8 ± 18.2	122.4 ± 17.1	118.4 ± 16.6	115.9 ± 18.7
**ScvO**_**2**_**(%)**	**1 (TEA)**	72.8 ± 8.4	75.2 ± 8.3	73.9 ± 10.8	74.4 ± 11.7	73.6 ± 13.2	71.6 ± 12.6	71.3 ± 9.6	69.1 ± 9.3	65.6 ± 12.9
	**2 (Control)**	81.6 ± 9.9	77.5 ± 9.6	79.4 ± 9.8	77.3 ± 9.4	70.8 ± 12.3	73.6 ± 12.2	73.4 ± 11.0	74.8 ± 11.5	72.0 ± 12.5
**Lactate(mmol/L)**	**1 (TEA)**	0.9 ± 0.3	0.8 ± 0.2	0.8 ± 0.2	0.9 ± 0.2	0.8 ± 0.2	0.8 ± 0.2	0.7 ± 0.2	0.7 ± 0.2	0.7 ± 0.2
	**2 (Control)**	0.9 ± 0.3	0.9 ± 0.4	0.9 ± 0.4	0.9 ± 0.4	0.8 ± 0.3	0.7 ± 0.2	0.6 ± 0.2	0.6 ± 0.2	0.6 ± 0.2
**Colloids (ml)**	**1 (TEA)**	59 ± 141	121 ± 193	397 ± 235	518 ± 203	676 ± 290	879 ± 367	1.088 ± 434	1.294 ± 460	1.594 ± 560
	**2 (Control)**	0 ± 0	50 ± 132	174 ± 209	388 ± 172	538 ± 156	768 ± 225	1.032 ± 171	1.382 ± 269	1.694 ± 420
**Cristalloids (ml)s**	**1 (TEA)**	757 ± 210	1047 ± 325	1.268 ± 403	1.518 ± 460	1.844 ± 548	2.347 ± 637	2.756 ± 748	3.232 ± 968	3.606 ± 1.103
	**2 (Control)**	621 ± 211	932 ± 292	1.271 ± 335	1.415 ± 363	1.829 ± 357	2.276 ± 370	2.788 ± 501	3.300 ± 663	0.3800 ± 794
**Urine (ml)**	**1 (TEA)**	297 ± 271	406 ± 370	579 ± 491	682 ± 552	1.012 ± 702	1.294 ± 833	1.571 ± 1.015	1.868 ± 1.166	2.188 ± 1.343
	**2 (Control)**	206 ± 228	335 ± 337	582 ± 559	762 ± 644	1.065 ± 748	1.424 ± 858	1.876 ± 1.013	2.376 ± 1.136	2.844 ± 1.305

Values for tissue oxygenation (tpo2 (mmHg)), microcirculatory flow (Flux (AU)), Heart rate (HR (min^-1^)), mean arterial pressure (MAP (mmHg)), cardiac index (CI (L/min)), systemic vascular resistance (SVRi (dynes*sec/cm^5^/m^2^)), stroke volume variation (SVV (%)), central venous pressure (CVP (mmHg)), arterial partial oxygen pressure (p_a_O_2_ (mmHg)), central venous oxygen saturation (ScvO2 (%)), lactate measured in arterial blood gas analysis (mmol/L), cumulative crystalloid infusion (ml), cumulative colloid infusion (ml) and urine output (ml). Data are presented as mean ± standard deviation. *represents statistically significant differenence between treatment groups (P <0.05) at time of measurement. M 0: before induction of the acute pancreatitis, M 1 and 2: after induction of the acute pancreatitis, M 2 to 8: during treatment interval. TEA, thoracic epidural anesthesia.

### Histopathologic examination

Histopathologic tissue examination revealed a lower severity of acute pancreatitis in Group 1 (TEA) compared to Group 2 (Control). Overall histopathologic pancreatitis score was 5.5 (3 to 8) (Group 1 (TEA)) versus 8 (5.5 to 10) (Group 2 (Control)) (*P* <0.001). Details on histopathologic scoring for acinar necrosis, fatty tissue necrosis, inflammation and edema in the pancreatic head are presented in Table 3.

**Table 3:** Histopathologic scoring for severity of acute porcine pancreatitis

**Histopathologic severity score of acute pancreatis**					
**Group**	Acinar necrosis*	Fatty tissue necrosis*	**Inflammation**	Edema*	Overall*
**Group 1 (TEA)**	2 (0–3)	1 (0–2)	2 (0–2)	1 (0–2)	5.5 (3–8)
**Group 2 (CONTROL)**	3 (0–3)	2 (1–3)	2 (1–3)	1.5 (0.5-3)	8 (5.5-10)
	Acinar necrosis	Fatty tissue necrosis (in relation to plane)	Inflammation (plasma cells, lymphocytes and granulocytes outside parechymal and fatty tissue)	Edema	
	0 nil	0 nil	0 nil	0 nil	
	1 <10 single necrosis/lobule	1 <1/3 of plane	1 loose infiltrates (≤30 cells/HPF)	1 intralobular edema	
	2 ≥10 single necrosis/lobule	2 ≥1/3 to <2/3 of plane	2 moderate infiltrates (30–99 cells/HPF)	2 interacinar edema, ≥2 lobules	
* *P* < 0.05	3 ≥1/3 of plane	3 ≥2/3 of plane	3 dense infiltrates (≥100 cells/HPF)	3 intercellular edema, ≥2 lobules	

*P <0.05 representing statistically significant difference. Data are presented as median (range); Pancreatitis Score: 0 (no pancreatitis) to 12 (severe pancreatitis); histopathologic pancreatitis score (0 to 12 points) including acinar necrosis (0 to 3) fatty tissue necrosis (0 to 3), inflammation (0 to 3) and Edema (0 to 3). HPF: High power field; TEA, thoracic epidural anesthesia.

### Hemodynamics and fluid balance

Hemodynamic data on macrocirculatory conditions did not present any significant differences during the entire intraopaerative period. Neither norepinephrine nor epinephrine was applied in either of the treatment groups. Detailed data on hemodynamics and fluid balance are presented in Table 2. The amount of fluids infused also did not differ between the treatment groups.

### Laboratory data

Neither arterial blood gas analysis during the treatment interval (M2 to M8) nor analysis of pancreatic amylase, liver enzymes, bilirubin, leucocyte count, lactate, and creatinine, from the blood samples taken in the postoperative observation period, presented any significant differences between the two treatment groups (*P* >0.05); (Table 4).

**Table 4:** Laboratory results of analysis of hemoglobin, leucocyte count, thrombocyte count, prothrombin time (PT), partial thromboplastin time (PTT), creatinine, aspartate-transaminase (AST), alanine-transferase (ALT), pancreatic amylase and total bilirubin

	**Group**	**M 0**	**M 1**	**M 2**	**M 3**	**M 4**	**M 5**	**M 6**	**M 7**	**M 8**	**Day 1**	**Day 2**	**Day 3**	**Day 4**	**Day 5**	**Day 6**	**Day 7**
**Hemoglobin (g/dl)**	**TEA**	8.8 ± 0.6	8.6 ± 0.6	8.2 ± 0.6	8.7 ± 0.7	9.1 ± 0.9	8.6 ± 0.5	8.6 ± 0.5	8.4 ± 0.7	8.2 ± 0.8	9.7 ± 0.8	9.6 ± 1.4	9.3 ± 1.2	9.2 ± 1.4	9.4 ± 1.2	9.3 ± 1.2	8.6 ± 1.4
	**Controll**	8.6 ± 0.5	8.7 ± 0.5	8.1 ± 0.6	8.5 ± 0.4	8.7 ± 0.5	8.4 ± 0.7	8.5 ± 0.9	8.0 ± 0.9	7.8 ± 0.6	9.5 ± 0.5	9.4 ± 1.2	9.4 ± 0.9	9.3 ± 0.4	9.3 ± 1.6	10.0 ± 0.7	9.5 ± 1.0
**Leucocytes (10**^**9**^**/L)**	**TEA**	9.2 ± 3.0	8.7 ± 3.9	10.8 ± 4.0	9.4 ± 4.5	11.9 ± 4.4	11.8 ± 3.4	11.0 ± 3.0	10.5 ± 3.4	9.5 ± 4.3	11.0 ± 4.0	15.1 ± 4.6	18.8 ± 6.8	17.0 ± 7.1	23.4 ± 7.4	24.1 ± 7.6	23.5 ± 7.2
	**Controll**	9.0 ± 3.4	11.3 ± 3.2	12.8 ± 5.3	11.9 ± 2.4	13.3 ± 3.1	12.8 ± 3.3	12.4 ± 3.8	11.6 ± 3.4	9.8 ± 4.3	11.4 ± 3.5	17.9 ± 4.6	18.2 ± 6.6	17.7 ± 3.9	21.5 ± 6.7	25.0 ± 2.4	21.2 ± 6.3
**Thrombocytes (10**^**9**^**/L)**	**TEA**	279 ± 97	196 ± 40	263 ± 72	176 ± 13	202 ± 38	191 ± 15	198 ± 19	186 ± 29	266 ± 97	356 ± 148	337 ± 103	380 ± 140	417 ± 174	445 ± 191	408 ± 178	403 ± 151
	**Control**	322 ± 97	240 ± 41	296 ± 100	251 ± 32	237 ± 42	250 ± 40	233 ± 20	211 ± 18	272 ± 100	366 ± 99	321 ± 94	333 ± 63	361 ± 100	423 ± 164	353 ± 175	408 ± 101
**PT (seconds)**	**TEA**	108 ± 9	110 ± 5	107 ± 8	112 ± 7	110 ± 6	113 ± 4	110 ± 7	110 ± 9	107 ± 10	94 ± 10	112 ± 8	109 ± 15	102 ± 12	98 ± 13	98 ± 11	94 ± 11
	**Control**	115 ± 8	108 ± 5	105 ± 23	107 ± 3	106 ± 5	104 ± 9	105 ± 5	101 ± 8	101 ± 11	97 ± 9	100 ± 31	112 ± 13	114 ± 14	104 ± 15	109 ± 11	98 ± 11
**PTT (seconds)**	**TEA**	89 ± 16	79 ± 15	85 ± 24	81 ± 27	86 ± 10	91 ± 26	93 ± 15	76 ± 4	92 ± 30	134 ± 22	103 ± 40	109 ± 22	79 ± 42	97 ± 48	107 ± 48	43 ± 25
	**Control**	84 ± 21	81 ± 18	86 ± 27	84 ± 27	82 ± 19	90 ± 30	75 ± 19	88 ± 19	98 ± 19	121 ± 25	111 ± 30	100 ± 33	87 ± 41	69 ± 17	79 ± 27	58 ± 14
**Creatinine (mg/dl)**	**TEA**	0.8 ± 0.1	0.8 ± 0.1	0.8 ± 0.1	0.8 ± 0.1	0.8 ± 0.1	0.8 ± 0.1	0.8 ± 0.1	0.8 ± 0.2	0.8 ± 0.1	0.8 ± 0.2	0.7 ± 0.2	0.8 ± 0.2	0.9 ± 0.4	1.0 ± 1.0	1.1 ± 1.3	1.2 ± 2.0
	**Control**	0.7 ± 0.1	0.8 ± 0.1	0.7 ± 0.1	0.8 ± 0.2	0.8 ± 0.2	0.8 ± 0.2	0.8 ± 0.2	0.8 ± 0.3	0.8 ± 0.1	0.7 ± 0.1	0.7 ± 0.2	0.6 ± 0.1	0.7 ± 0.1	0.7 ± 0.1	0.7 ± 0.2	0.7 ± 0.1
**AST (U/L)**	**TEA**	38 ± 13	42 ± 22	38 ± 14	45 ± 18	48 ± 20	47 ± 19	51 ± 22	52 ± 18	60 ± 21	348 ± 220	116 ± 62	54 ± 25	40 ± 19	32 ± 15	30 ± 14	41 ± 48
	**Control**	34 ± 11	40 ± 12	40 ± 15	40 ± 13	43 ± 10	46 ± 13	51 ± 8	51 ± 13	58 ± 19	319 ± 116	86 ± 27	45 ± 12	33 ± 5	30 ± 4	29 ± 12	32 ± 9
**ALT (U/L)**	**TEA**	77 ± 15	80 ± 6	63 ± 13	77 ± 9	76 ± 5	70 ± 9	66 ± 4	63 ± 8	50 ± 8	111 ± 30	105 ± 25	93 ± 24	77 ± 24	74 ± 19	66 ± 16	49 ± 15
	**Control**	76 ± 13	70 ± 12	63 ± 11	62 ± 9	61 ± 11	55 ± 8	55 ± 9	47 ± 7	46 ± 10	115 ± 28	105 ± 21	88 ± 15	82 ± 9	81 ± 13	71 ± 13	50 ± 12
**Amylase (U/L)**	**TEA**	2155 ± 368	2606 ± 103	2388 ± 509	2808 ± 137	2960 ± 280	2895 ± 250	3011 ± 187	3045 ± 319	3153 ± 965	8726 ± 3736	8196 ± 4195	6745 ± 4591	4316 ± 2923	3966 ± 2930	3909 ± 3319	3225 ± 3886
	**Control**	1971 ± 432	2165 ± 476	2249 ± 814	2278 ± 556	2479 ± 541	2567 ± 699	2876 ± 953	2733 ± 978	3261 ± 1296	10186 ± 5195	9001 ± 6610	8472 ± 5937	8109 ± 7347	6726 ± 5501	10129 ± 6670	7926 ± 6196
**Bilirubin (mg/dl)**	**TEA**	0.2 ± 0.0	0.2 ± 0.0	0.2 ± 0.1	0.2 ± 0.0	0.2 ± 0.0	0.2 ± 0.0	0.2 ± 0.0	0.2 ± 0.0	0.2 ± 0.0	0.4 ± 0.2	0.2 ± 0.0	0.2 ± 0.0	0.2 ± 0.0	0.2 ± 0.1	0.2 ± 0.1	0.2 ± 0.1
	**Control**	0.2 ± 0.1	0.2 ± 0.0	0.2 ± 0.1	0.2 ± 0.0	0.2 ± 0.0	0.2 ± 0.0	0.2 ± 0.0	0.2 ± 0.0	0.2 ± 0.0	0.3 ± 0.1	0.2 ± 0.0	0.2 ± 0.0	0.2 ± 0.0	0.2 ± 0.0	0.2 ± 0.0	0.2 ± 0.0

M0 to M8: during intraoperative setting. Day1 to Day7: results of blood samples taken in the postoperative observation period. TEA, thoracic epidural anesthesia.

## Discussion

This study analyzed the effects of TEA in SAP in an experimental setting. We found that TEA improved survival as well as pancreatic microcirculation and tissue oxygenation resulting in reduced histopathologic tissue damage. It is the first study assessing the effects of TEA during controlled hemodynamic conditions comparable to an intensive care setting. This is a necessary prerequisite to reliable assessment of microcirculatory dysfunction and tissue oxygenation.

The rationale for the use of TEA in acute pancreatitis is that intestinal and hepatic perfusion is regulated by sympathetic and parasympathetic nerves. In healthy subjects the regulation of blood flow is optimized for maintaining metabolic stability. During resting conditions the sympathetic tone is low and blood flow is mainly regulated by vagal tone activity. An increasing sympathetic activity resulting in an intestinal vasoconstriction and consequently reduced blood flow has been shown in acute stress and pain [28]. This is also true for systemic inflammation where the microcirculation is affected [22,36,37]. The effects of TEA have been analyzed in different experimental models. Adolphs and co-workers were able to detect an improved microcirculation after TEA during hemorrhage-induced impairment of intestinal perfusion [38]. Additionally, there are several studies in animals suffering from systemic inflammation demonstrating the benefits of using TEA. Models using cecal ligation to induce severe systemic inflammation demonstrated an improvement of sepsis-induced alterations of hepatic blood flow as well as improved mucosal microcirculation in rats [24,26]. In a model of systemic inflammation during endotoxemia in an ovine model as well as in the rat, superior renal perfusion as well as an attenuation of impairment of gastrointestinal organ perfusion and improved microvascular mucosa perfusion could be demonstrated [22,24,25]. A study by Lauer *et al*. demonstrated that TEA also improved pulmonary endothelial integrity in hyperdynamic sepsis [39].

Data regarding TEA and pancreatitis are rare. Up to now, only two feasibility studies using TEA in acute pancreatitis in humans have been published. In the first trial, it was shown that the use of TEA is safe and the rate of complications is low, although no control group was investigated. In the second trial, TEA was found to be superior in terms of pain management [40,41]. Overall, no clinical data concerning the therapeutic effect of TEA in SAP, especially on improvement of outcome and survival, are available. Since there is enormous experience in the clinical use of TEA in humans for major abdominal and thoracic surgery, as well as for acute and chronic pain management, the implementation of TEA in the early treatment of SAP appears possible if no contraindications are present. Nonetheless, the idea of also using TEA in systemic inflammation and sepsis is still controversial [42]. Up to now, three experimental trials analyzing the impact of TEA in acute pancreatitis in rats have been published. In a setting similar to ours, Demirag and co-workers for the first time demonstrated an improved pancreatic microcirculation also using laser Doppler flowmetry as well as reduced histopathologic damage in a small series in the rat. However, no survival data were available [27]. Freise *et al*. were the first to analyze the impact of TEA on survival in an experimental model of SAP in the rat. Besides improved seven day survival they found an increased capillary perfusion and lower Inflammation, while no significant difference regarding histopathologic damage was present [43]. Another study by Freise and coworkers analyzed the impact of TEA on the liver in acute pancreatitis. Here, a reduction of the vasoconstriction of the sinusoids and reduced apoptosis was found [28]. These data are completely in accordance with our findings that applying TEA resulted in improved microcirculation and oxygenation of the pancreas and, finally, better survival as well as less histopathologic damage in SAP. Moreover, in our study the effects of TEA on survival could be evaluated in a model in which TEA was applied continuously over a period of seven days similar to a clinical setting, since animals were equipped with an automated infusion pump. To date, for practical reasons no direct assessment of pancreatic microcirculation exists in awake animals. Nonetheless, the effect of sympathetic block by TEA has also been demonstrated for awake animals [44]. In the animals included in this study no obvious signs of a motor block were present. Unfortunately, there is no reliable and validated method to assess analgesic effects and a sensory block, such as a pain scoring system, available in pigs. Although for reasons of the experimental setup no direct evaluation of the sympathicolytic effect was carried out, the correct positioning of the epidural catheter was verified at the end of the observation period by performing another epidurogram. Therefore, we can only postulate that the epidural catheter was in the correct position and that due to the application of bupivacaine a sympathetic block and a sensory effect were present and that the detected effects of TEA on pancreatic microcirculation and tissue oxygenation during the intraoperative treatment period are present during the entire protocol, thereby contributing to the superior survival of the TEA group. Our findings are, therefore, in accordance with the aforementioned theoretical considerations and are in line with the positive effects observed in other models of systemic inflammation using endotoxemia or bowel perforation. What has to be considered is that, overall, not only pancreatic microcirculation but also other aspects, such as improved intestinal mucosal perfusion with less gut barrier dysfunction and improved liver perfusion, potentially might have contributed to improved survival in our setting, especially since the liver also is involved in a multitude of physiologic processes and decisively contributes to the host’s immune reaction in sepsis and inflammation [28].

Some other aspects need to be taken into consideration. With a view to the evaluation of effectiveness of the treatment of SAP, it is important that a model is used that closely mimics the clinical situation with a high mortality in SAP where the severity of the pancreatitis ensures that some animals survive but also that some animals die without treatment. In our setting a model of intraductal injection of bile acid in the main pancreatic duct followed by closure was used. The model closely represents the clinical situation of acute biliary pancreatitis caused by obstruction of the papilla by bile stones and has been established in previous trials [9,45-47]. Another advantage of the model is that it allows a very high standardization, using standardized dosages and infusion pressures unlike other models that induce SAP by ischemia, hypotension or ingestion of alcohol [48,49].

Another consideration is that in our study we cannot explicitly demonstrate the sympathetic block. Measurements of the arterial plasma levels of epinephrine and norepinephrine were not carried out and also thermal imaging was not possible because of the experimental setup. When looking at hemodynamics we also could not find a significant impact due to the onset of TEA. The level chosen at Th 7/8 for TEA will not result in relevant vasodilation of capacitance vessels; neither should there be a relevant effect on the nervi accelerantes. Therefore, according to our understanding, it is not surprising that the onset of TEA did not present with significant changes and result in significant differences of hemodynamic parameters.

In addition to the data presented, we analyzed the effects of TEA in a small series of healthy animals as well, where no experimental induction of pancreatitis was carried out. In one group only surgical preparation was carried out, while in the other group surgical preparation and TEA were performed. Most importantly, it was shown that the pancreatitis is not caused by the experimental setting (except the intraductal injection of bile acid), surgical trauma or anesthesia or TEA. No differences regarding pancreatic microcirculation and tissue oxygenation were detected and no sign of pancreatitis or inflammation was found in the histopathologic examination.

An issue worth discussing is the interval between induction of pancreatitis and the beginning of treatment. In our study, the interval chosen was rather short. However, to our understanding this seems to be adequate, because the direct intraductal injection of bile acid induces an acute pancreatitis within a few minutes, which is much faster than acute biliary pancreatitis found in the clinical setting [50,51]. In our experimental setting, SAP was observed macroscopically in all animals prior to the beginning of therapeutic intervention. If the interval between induction and beginning of the treatment is too long the effect of improvement of the pancreatic microcirculation may not occur when fulminate necroses are already present, as the rationale for the treatment approach is to improve microcirculatory perfusion and thereby save not yet irreversibly injured tissue from infarction and necrosis [52,53]. This should not be forgotten and similar to approaches for early goal-directed hemodynamic stabilization by fluid therapy in systemic inflammation, it probably holds true that the treatment interval needs to be short and TEA should be commenced as early as possible.

Concerning the animal model, pigs were chosen since in smaller animals no adequate hemodynamic monitoring and therapy is possible. This is essential to rule out macrocirculatory effects on pancreatic microcirculation as potential confounding factors influencing the estimation of the effects of TEA in our trial. The combination of invasive intrapancreatic measurement of tissue oxygenation and microcirculation by microvascular blood flow not only allows a reliable assessment of the pancreatic microcirculatory conditions, it also allows evaluation of the amount of oxygen reaching the end organ. Although a substantial benefit of TEA in SAP was detected in our animal model, the direct transfer of the results to humans is not proven. The conception of the trial with a seven-day observation period allowed evaluation of the outcome for SAP and in this regard represents a good model closely mimicking the clinical situation and an important basis for potential further prospective randomized clinical trials.

Another fact is that measurement of pancreatic tissue oxygenation and microcirculation was limited to a period of only six hours. For this reason, we cannot make a definite statement on the effects of TEA on pancreatic microcirculation and tissue oxygenation in the later course. Nonetheless, the effects and differences are present at an early stage and TEA led to a significant improvement in survival and less histopathologic damage. This strengthens the assumption of our hypothesis that TEA has a relevant therapeutic effect in SAP.

## Conclusions

In conclusion, our data suggest that application of TEA resulted in improved survival and led to enhanced pancreatic microcirculation and tissue oxygenation resulting in reduced histopathologic damage in a model of severe acute porcine pancreatitis.

## Key messages

• The use of epidural anesthesia in systemic inflammation is still controversial.

• The results of our study suggest that there is a relevant improvement in pancreatic microcirculation and tissue oxygenation due to the use of TEA in SAP.

• We could demonstrate that this improvement due to TEA resulted in less histopathologic damage and improved survival.

• Further evaluation is required to transfer these promising results into clinical practice.

## Abbreviations

ALT: Alanine transaminase; ANOVA: Analysis of variance; AST: Aspartate transaminase; Fet: End tidal fraction; FiO2: Inspiratory oxygen fraction; HPF: High power field; MAP: Mean arterial pressure; PT: Prothrombin time; pTT: Partial thromboplatin time; SAP: Severe acute pancreatitis; TBIL: Total bilirubin; TEA: Thoracic epidural anesthesia; tpO2: Tissue oxygen tension.

## Competing interests

The authors declare they have no competing interests.

## Authors’ contributions

KB, CT, DR and OM made substantial contributions to conception and design, analysis and interpretation of data and have been involved in drafting and revising the manuscript. AG, TS and JI were involved in analysis and interpretation of data and in drafting and revising the manuscript critically for important intellectual content. LT, JS, WB and LH participated in the acquisition of data and execution of the experimental protocol. AH carried out the histopathologic examination. All authors read and approved the final manuscript.
